# Serine phosphorylation regulates paxillin turnover during cell migration

**DOI:** 10.1186/1478-811X-4-8

**Published:** 2006-11-22

**Authors:** Nancy Abou Zeid, Ana-Maria Vallés, Brigitte Boyer

**Affiliations:** 1 Institut Curie, CNRS UMR146, Centre Universitaire, Orsay, France; 2 Laboratoire de Génétique Moléculaire du Développement INSERM UR784, Département de Biologie, Ecole Normale Supérieure, 46 rue d'Ulm 75005, Paris, France

## Abstract

**Background:**

Paxillin acts as an adaptor protein that localizes to focal adhesion. This protein is regulated during cell migration by phosphorylation on tyrosine, serine and threonine residues. Most of these phosphorylations have been implicated in the regulation of different steps of cell migration. The two major phosphorylation sites of paxillin in response to adhesion to an extracellular matrix are serines 188 and 190. However, the function of this phosphorylation event remains unknown. The purpose of this work was to determine the role of paxillin phosphorylation on residues S188 and S190 in the regulation of cell migration.

**Results:**

We used NBT-II epithelial cells that can be induced to migrate when plated on collagen. To examine the role of paxillin serines 188/190 in cell migration, we constructed an EGFP-tagged paxillin mutant in which S188/S190 were mutated into unphosphorylatable alanine residues. We provide evidence that paxillin is regulated by proteasomal degradation following polyubiquitylation of the protein. During active cell migration on collagen, paxillin is protected from proteasome-dependent degradation. We demonstrate that phosphorylation of serines 188/190 is necessary for the protective effect of collagen. In an effort to understand the physiological relevance of paxillin protection from degradation, we show that cells expressing the paxillin S188/190A interfering mutant spread less, have reduced protrusive activity but migrate more actively.

**Conclusion:**

Our data demonstrate for the first time that serine-regulated degradation of paxillin plays a key role in the modulation of membrane dynamics and consequently, in the control of cell motility.

## Background

Cell migration is a cyclic multistep event that involves cell polarization and extension of membrane protrusions at the leading edge. At the front of the protrusion, cell-substratum adhesive complexes are formed providing traction points for translocation. Adhesive complexes at the cell rear are then released as the cell continues to move forward [1].

Integrins are cell-surface adhesion receptors that link the extracellular matrix (ECM) to the intracellular actin cytoskeleton [2]. Integrins are heterodimeric receptors that interact with numerous structural and signaling molecules to form large protein complexes, the focal adhesions. Upon binding to ECM elements, integrins cluster and interact with an array of structural and signaling molecules to form large protein complexes, the focal adhesions. These structures are spatially regulated during cell migration as they assemble at the leading edge to stabilize new membrane protrusions and disassemble at the rear of the cell to allow cell detachment [3].

The assembly process of focal adhesions at the leading edge has been extensively examined, leading to the conclusion that they are highly dynamic. Their molecular composition varies over time [4,5], thereby regulating membrane protrusive activity and cell movement. For instance, fast movement of the lamellipodium has been associated with focal complexes containing relatively low levels of paxillin whereas their slower movement with focal complexes rich in paxillin [5].

Compared with the process of formation, less is known about adhesion breakdown. Adhesive complexes are disassembled at the rear and at the base of dynamic protrusions [4] in polarized migrating cells. Proteolysis of focal adhesion components has been proposed to regulate the protein content of adhesive complexes resulting in the control of focal adhesion turnover and cell migration. Calpains, cysteine proteases which localize to adhesive structures [6], have been shown to cleave several focal adhesion proteins, such as focal adhesion kinase (FAK) [7], talin [8] and paxillin [9-11]. It is now accepted that these proteases play an essential role in regulating focal adhesion dynamics, membrane protrusion stability and cell migration.

The ubiquitin-dependent proteasomal degradation is another proteolytic pathway that has been implicated in the control of focal adhesion turnover and cell migration. Proteasomal degradation of FAK was shown to inhibit cell migration [12]. Similarly, ubiquitylation of paxillin was shown to affect its localization to focal adhesions and to inhibit cell migration [13].

Paxillin is a 68 kD adapter protein that localizes to focal adhesions. Paxillin contains many protein-protein interaction domains: five LD motives (Leucine-rich domains) in the amino-terminal part and four LIM domains (double zinc fingers) in the carboxy-terminal part. LIM domains mediate binding to protein tyrosine phosphatase-PEST (PTP-PEST) [14,15], α- and γ-tubulin [16]. LIM3 is responsible for proper targeting of paxillin to focal adhesions [17]. LD motifs mediate interactions with an array of partners, including FAK [17] and vinculin [18].

Regulation of paxillin by phosphorylation is well documented [19]. Tyrosine residues are phosphorylated in response to extracellular signals and serve as docking sites for protein interactions. Phosphorylation of tyrosines 31 and 118 provides binding sites for the SH2 domain of Crk and this interaction is necessary for cell migration [20]. Serine phosphorylation of paxillin is also a prevalent posttranslational modification that has been extensively examined. Paxillin serine phosphorylation is increased by cell adhesion on fibronectin [21] or vitronectin [22]. In contrast, during cell mitosis, which is associated with an absence of adhesive complexes, paxillin serine phosphorylation coincides with its degradation [11]. ERK and GSK-3 have been reported to phosphorylate serines 126 and 130, respectively, leading to the relocalization of paxillin from focal adhesions to the cytosol [23,24], while serine 178 phosphorylation by JNK is necessary for cell migration [25]. It appears therefore that the phosphorylation of paxillin at specific serine/threonine residues could regulate its activity and biological functions. A function has still to be attributed to the phosphorylation of serines 188 and 190 of paxillin, the two major phosphorylation sites in response to cell adhesion to an extracellular matrix.

Because paxillin is a focal adhesion adaptor protein, it is possible that specific serine phosphorylation events control its degradation rate, as already suggested [11], thereby influencing cell movement through the regulation of focal adhesion dynamics. The implication of paxillin in focal adhesion turnover has been recently demonstrated. Horwitz and coworkers showed that the rate of focal adhesion disassembly is slower in cells expressing several phosphorylation mutants or domain-deleted forms of paxillin compared to cells expressing wild-type paxillin [26].

In this report, we found that paxillin could be ubiquitylated and degraded by the proteasome. Moreover, we show that substrates such as collagen that support cell migration prevented paxillin degradation. Using a S188/190A phosphorylation mutant of paxillin, we were able to demonstrate that phosphorylation of S180/S190 was required for the increased stability of paxillin on collagen. We also showed that paxillin S188/190A double mutant impaired membrane protrusion dynamics as well as cell migration and spreading without affecting cell adhesion on collagen.

## Results

### Paxillin ubiquitin-dependent proteasomal degradation is regulated by collagen

**Figure 1:**
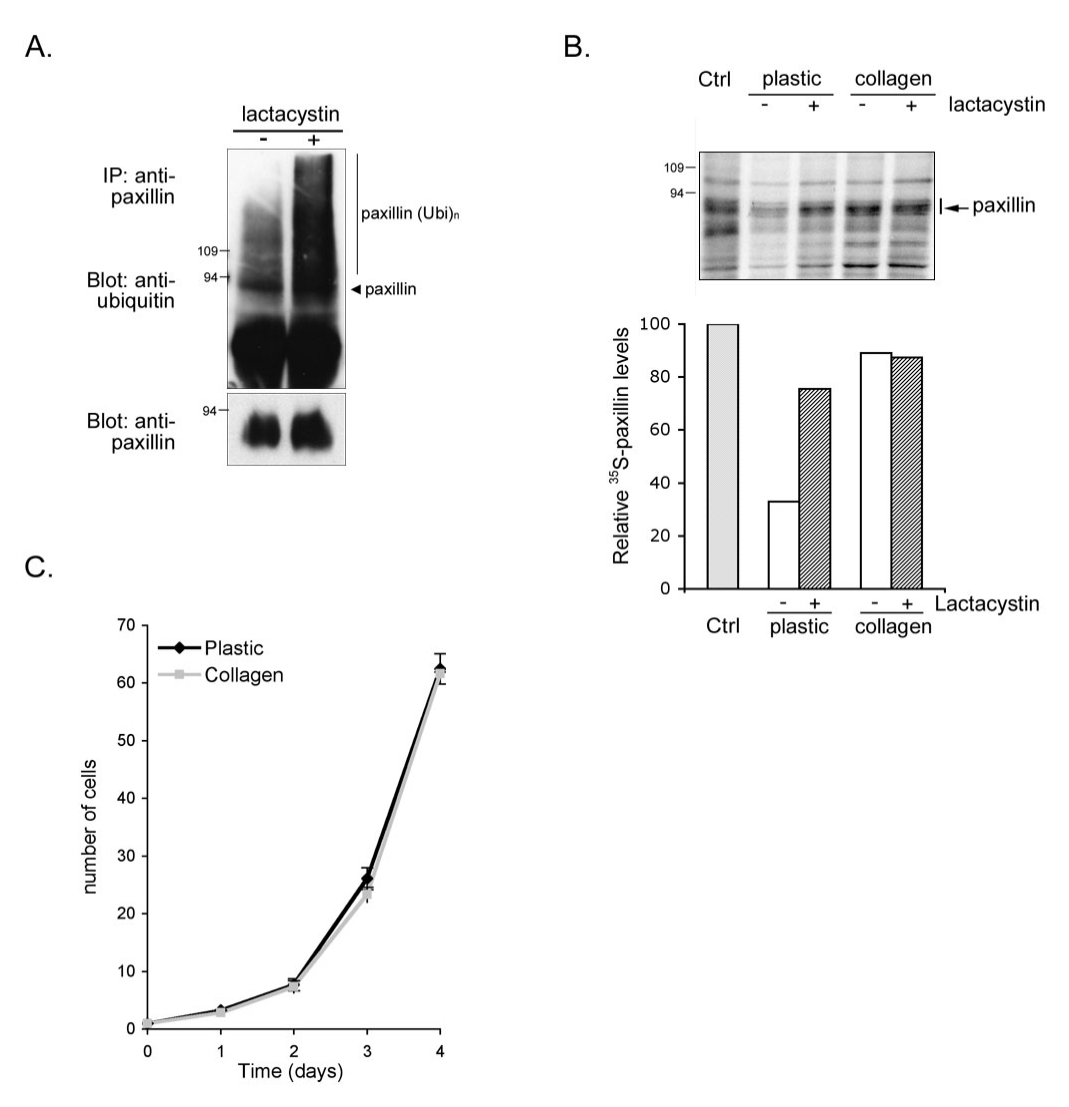
**Paxillin proteasomal degradation is regulated by collagen**. A) NBT-II cells plated for 18 h on plastic dishes were treated with the proteasome inhibitor lactacystin at 1 μM for 4 h. Cell lysates were immunoprecipitated with anti-paxillin antibody and immunoblot analysis was done with anti-ubiquitin (upper panel) or anti-paxillin antibody (lower panel). B) NBT-II cells were pulse-chased on plastic or collagen-coated dishes for 18 h and cell lysates were subjected to paxillin immunoprecipitation as described in Materials and Methods. When indicated, 1 μM lactacystin was added to the cells during the chase period. The lower panel is a quantification of the bands corresponding to ^35^S-labeled paxillin. The ^35^S-paxillin levels were calculated relatively to the density of the paxillin band immunoprecipitated from cells at the beginning of the radioactive chase (control lane). C) NBT-II cells cultured for the indicated times on plastic or collagen-coated dishes were trypsinized and counted using a hemocytometer. Values represent the mean of three independent experiments ± SEM.

The turnover of focal adhesions and their components is known to play a crucial role during cell locomotion events [26]. To determine whether paxillin, a docking protein of focal adhesion sites, is subjected to proteasomal degradation, NBT-II cells were treated for 4 h with lactacystin, a selective 26S proteasome inhibitor, prior to cell lysis. Equivalent amounts of proteins from total cell extracts were immunoprecipitated with an anti-paxillin antibody and subjected to immunoblot with an anti-ubiquitin antibody. Ubiquitylation of paxillin was detected as a smear of high molecular weight bands, corresponding to polyubiquitylated species (Fig. 1A). Treatment with lactacystin strongly increased the ubiquitylated bands, indicating that paxillin is degraded by the proteasome following its polyubiquitylation.

We next wished to determine whether paxillin degradation could be modified under conditions allowing cell migration. Since NBT-II carcinoma cells are induced to migrate on collagen substrates [30], cells were labeled with ^35^S-methionine/cysteine mix and chased on plastic or collagen for 18 h in the presence or absence of lactacystin. Uncoated plastic plates were used as a substrate that does not induce cell migration. As shown in Figure 1B, paxillin protein levels significantly decreased after 18 h chase. Quantification of the radioactive bands migrating with the apparent molecular weight of paxillin indicated an approximate reduction of 70% of the newly synthesized molecules during the incubation time. This decrease was mostly due to proteasomal degradation since it could be prevented by treatment of cells with lactacystin added during the entire chase. In contrast, paxillin levels were not reduced during the chase period on collagen substrate. As a consequence, lactacystin had no obvious effect on paxillin stability under those conditions. The additional bands that are visible below and above the apparent size of paxillin probably represent proteins that co-precipitated with the adaptor protein paxillin. The differences in paxillin half life in cells plated on collagen versus plastic were apparently specific to paxillin as other proteins, such as tubulin, did not exhibit similar variations (data not shown). These experiments indicate that the half-life of paxillin of approximately 7 h in stationary cells on plastic is noticeably increased in motile cells on collagen, suggesting that collagen prevents paxillin proteasomal degradation.

It is well established that ECM protects cells from programmed cell death. To ensure that the accelerated degradation of paxillin in NBT-II cells plated on plastic as compared to collagen was not due to an increase in cell death on plastic, the number of cells plated on each substrate was evaluated over a 5-day period (Fig. 1C). No difference in cell numbers on plastic and collagen was observed. These results suggest that collagen-induced reduction of paxillin degradation is linked to induction of cell motility rather than to protection from cell death.

### Phosphorylation of serines 188/190 prevents paxillin from degradation

Serine/threonine phosphorylation events are known to regulate ubiquitin-dependent proteasomal degradation of several proteins [31-33]. Paxillin, originally identified as a tyrosine phosphorylated protein [34,35], is also heavily phosphorylated on serine/threonine residues [11,21,23-25,36-39]. Phosphorylation of specific serine residues of paxillin has been shown to be implicated in cytoskeleton rearrangement and cell motility [23,25,37]. Interestingly, two recent reports have proposed a possible link between serine phosphorylation-dependent degradation of paxillin and cell movement [25,37]. Two major sites of serine phosphorylation are residues 188/190, previously shown to be heavily phosphorylated upon cell adhesion to ECM [21]. However, the role of these modifications remains undefined.

**Figure 2:**
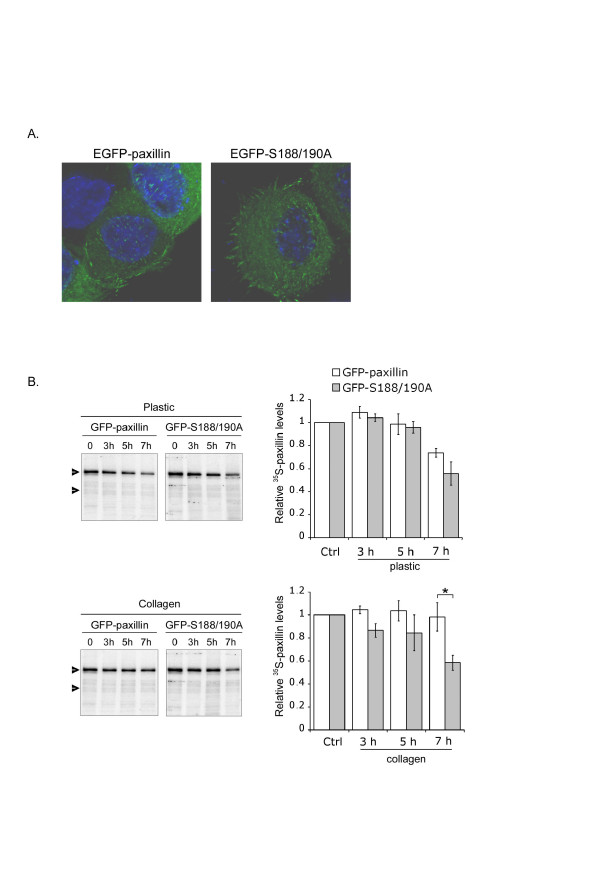
**Collagen protects EGFP-S188/190A from degradation**. A) NBT-II cells transfected with EGFP-paxillin or EGFP-S188/190A were plated on glass cover-slips for 18 h prior to fixation. The localization of the fusion proteins was observed by EGFP-fluorescence microscopy. B) NBT-II cells expressing wild type or mutant paxillin were pulse-chased on plastic or collagen-coated dishes for the indicated times. Cell lysates were immunoprecipitated with paxillin antibody prior to SDS-PAGE followed by autoradiography (left panels). The upper arrowhead points to EGFP-paxillin proteins bands and the lower arrowhead to endogenous paxillin bands. By densitometric analyses, mean exogenous ^35^S-paxillin levels were calculated relatively to the density of the paxillin band in the control lane. Means and SEM from 3 independent experiments were then calculated. The Student's *t *test was used to evaluate the data (right panels). *, p < 0.05.

To examine the potential role of serines 188/190 phosphorylation in paxillin degradation, we constructed an EGFP-tagged paxillin mutant in which S188/S190 were mutated into unphosphorylatable alanine residues (S188/190A). GFP fluorescent dots were clustered at focal adhesions at the periphery of cells transfected with either forms of paxillin, indicating that the S188/190A paxillin mutant localized to focal adhesions as the wild-type EGFP-paxillin (Fig. 2A). By pulse chase analysis, we evaluated the stability of the mutant form as compared to wild type paxillin. ^35^S-labelled transfected cells plated on either plastic or collagen were chased for up to 7 h and the stability of exogenous paxillin was determined as described before. In agreement with the data obtained after the 18 h chase, the half-life of exogenously expressed wild type or mutant paxillin in cells plated on plastic was approximately 7 h (Fig. 2B). When cells were plated on collagen, the levels of wild type paxillin were sustained during the chase period, as already noticed (Figure 1B). In sharp contrast, collagen did not stabilize EGFP-S188/190A paxillin, as the half-life of the mutant protein of cells plated on collagen was similar to that of cells on plastic (~7 h). These observations indicate that phosphorylation of residues S188/S190 regulates paxillin degradation and suggest furthermore that this phosphorylation event is downstream of collagen signaling.

### Mutations on serines 188/190 do not affect cell adhesion but inhibit cell spreading

**Figure 3:**
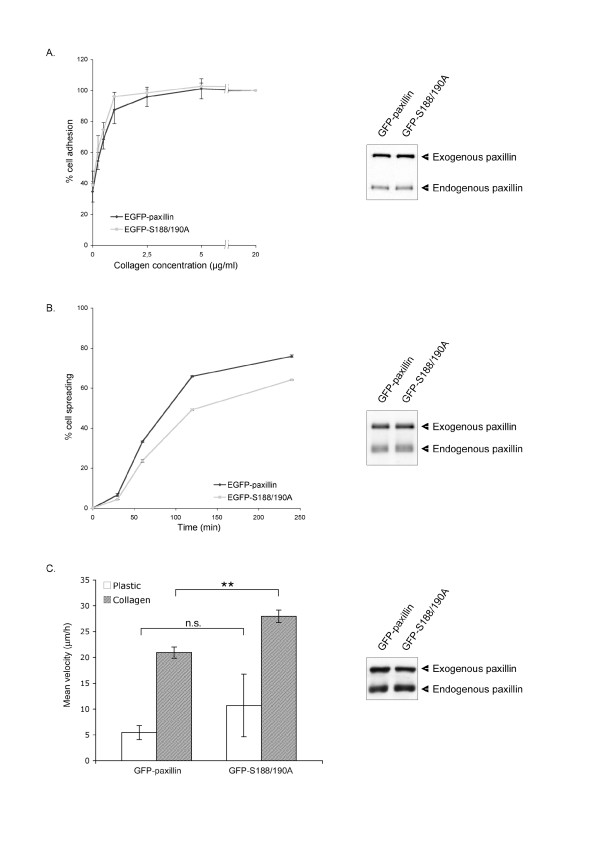
**Cells expressing EGFP-paxillin or EGFP-S188/190A paxillin have similar adhesion but different spreading and migration rates on collagen**. A) NBT-II cells transfected with EGFP-paxillin or EGFP-S188/190A were allowed to adhere on dishes coated with collagen at the indicated concentrations for 1 h at 37°C. Absorbance values were obtained by crystal violet uptake assay and expressed as percent of maximal adhesion. Values represent the mean of three independent experiments carried out in triplicate ± SEM (left panel). Expression of endogenous and transfected paxillin is shown on the right panel. B) Cell spreading was quantified by allowing cells to adhere onto collagen coated plates (10 μg/ml) for the indicated times. The percentage of spread cells among the GFP-positive population was then calculated for each time point. Values represent the mean of three independent experiments carried out in triplicate ± SEM (left panel). Expression of endogenous and transfected paxillin is shown on the right panel. C) NBT-II cells transfected with either plasmids were plated on plastic or collagen-coated plates (10 μg/ml) for 4 h. Cell motility was evaluated by tracking individual cells for 10 h using the Metamorph software. Mean migration rate of cells is expressed as μm/h. Values represent the mean of three independent experiments ± SEM (left panel). Expression of endogenous and transfected paxillin is shown on the right panel. **, p < 0.01

To investigate the significance of the increase in paxillin stability induced by collagen, we analyzed the functional effects of EGFP-S188/190A paxillin. We first determined the kinetics of adhesion on collagen of cells transiently expressing the EGFP-S188/190A mutant form as compared to wild type paxillin. Cells were plated on increasing concentrations of collagen for 1 h, fixed, and the number of adherent cells quantified by a colorimetric assay. Transfection efficiencies were estimated visually at 90% for both constructs and their expression were similar, as assessed by immunoblot analysis (Fig 3A, right panel). No significant difference in the adhesion rates of cells expressing either EGFP-paxillin or EGFP-S188/190A was observed, both cell types achieving half-maximal adhesion at 0.5 μg/ml, with maximal adhesion at 2.5 μg/ml. These results are in agreement with a previous report showing that the S188/190A paxillin double mutant has no effect on the adhesion rate of CHO-K1 cells on fibronectin [36].

We next analyzed the effect of the S188/190A paxillin mutant on cell spreading on collagen. Cells expressing EGFP-paxillin or EGFP-S188/190A were plated on collagen (10 μg/ml) for 30, 60, 120 and 240 min, and the number of EGFP-positive spread cells estimated after fixation. Cells expressing the S188/190A mutant spread slower than those expressing wild-type paxillin (Fig. 3B). Two hours after plating cells on collagen, there was a reproducible and significant 15% decrease in the ratio of spread cells expressing EGFP-S188/190A paxillin as compared to EGFP-paxillin (p < 0.001). These results suggest that phosphorylation on serines 188/190 contributes to cell spreading on collagen but not to cell attachment. Furthermore, these data argue for a dominant negative effect of S188/190A paxillin double mutation. The presence of endogenous paxillin may minimize the inhibition due to the mutant molecule. Alternatively, the moderate inhibition is comprehensible, considering that paxillin S188/S190 phosphorylation is only one element of the signaling pathway(s) leading to cell spreading.

### Mutations on serines 188/190 enhance random cell migration on collagen

Serine phosphorylations of paxillin have been shown to be involved in cell migration [25,40]. To determine whether the S188/190A mutant of paxillin affected random cell migration induced by collagen, we used time-lapse videomicroscopy to follow the movement of isolated cells expressing EGFP-tagged paxillin during 10 h. Using Metamorph software, we quantified the migration rate as the total distance traveled by one cell divided by the time of recording. On non-permissive substrates such as plastic plates, the absence of active cell migration of cells expressing the S188/190A paxillin mutant (10.71 μm/h ± 6.07) was in the same range as that of cells expressing EGFP-paxillin (5.46 μm/h ± 1.37). Mutations of paxillin S188/S190 into unphosphorylatable residues significantly increased migration on collagen (27.98 μm/h ± 1.21) as compared to cells expressing wild type paxillin (20.95 μm/h ± 1.09). Therefore, phosphorylation of residues S188/S190 is negatively implicated in cell migration on collagen.

### Mutations on S188/S190 inhibit membrane protrusion dynamics

**Figure 4:**
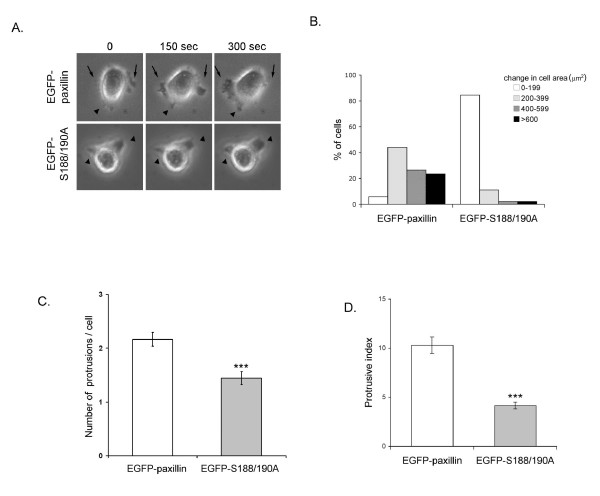
**Expression of EGFP-S188/190A paxillin inhibits membrane protrusion dynamics**. NBT-II cells expressing either paxillin constructs were plated on collagen-coated plates (10 μg/ml) for 4 h. Images of GFP-positive cells were taken on a fluorescence inverted microscope every 15 sec for 5 min. A) Representative images of cells expressing wild type or mutant paxillin used in protrusion analysis (performed as described in Materials and Methods) were taken from time-lapse series. Arrowheads point to stable and arrows to dynamic protrusions. Note the number of protrusions and their lability in EGFP-paxillin cell compared to cell expressing mutant paxillin. B) Protrusiveness was calculated as the net change in cell area (μm^2^). The distribution of protrusiveness within the cell population is shown. C) The mean number of protrusions per cell ± SEM was calculated by adding the cell extensions observed in all images and dividing the resulting figure by the number of images in the movie. D) Membrane protrusion index, which was quantified as described under Materials and Methods, represents the mean activity of a single protrusion. Data are the average of three independent experiments ± SEM. ***, p < 0.001.

Because mutations of S188/S190 into alanine residues accelerated NBT-II cell migration on collagen, we wanted to examine whether this mutation affected the cell protrusive activity, which plays an essential role in generating cell migration. Cells transiently expressing EGFP-paxillin or EGFP-S188/190A were plated on collagen-coated 6-well plates for 4 h. Using time-lapse videomicroscopy, light-transmission images of cells were taken every 15 sec for 5 min (Fig. 4A). The change in cell area of protrusions over time was calculated as described under Materials and Methods and the frequency distribution in the cell population shown in Figure 4B. Most cells expressing wild type paxillin had a change in cell area greater than 200 μm^2^, with a homogenous distribution above that value. In sharp contrast, the majority of cells expressing S188/190A paxillin were found in the group of low protrusive activity ( < 200 μm^2^). Because the reduction of protrusiveness observed in cells expressing the paxillin mutant could be due to a decrease either in the number of extended protrusions or in the dynamic of these protrusions, we analyzed two additional parameters. First, we calculated the total number of protrusions extended by each GFP-positive cell during the 5 min period of acquisition (Fig. 4C). Given the kinetics of extension/retraction of protrusions in NBT-II cells (data not shown), the 15 sec interval between two successive images was short enough to include unstable as well as stable protrusions. By comparing the number of membrane extensions in cells expressing wild type versus mutant forms of paxillin during the 5 min recording, we observed that expression of the S188/190A paxillin mutant significantly reduced the number of protrusions emitted by cells (Fig. 4C). We then evaluated the mean activity of protrusions in cells expressing either EGFP-S188/190A or EGFP-paxillin (Fig. 4D). Cells expressing mutant paxillin had a 60% decrease in collagen-induced protrusive activity as compared to cells expressing wild type paxillin. Collectively, these two sets of data indicate that the reduction in cell protrusive activity was due to a reduction both in the number of protrusions and in individual protrusion dynamics. These results also indicate that serine phosphorylation of residues 188/190 is necessary for membrane protrusion extension/retraction.

## Discussion

In this study, we provide evidence that paxillin levels can be regulated by proteasomal degradation following polyubiquitylation of the protein. On collagen, which sustains active cell migration of NBT-II cells, paxillin is protected from proteasome-dependent degradation leading to an increase in its half-life. In addition, we demonstrate that phosphorylation of serine residues 188 and 190 is necessary for the protective effect of collagen. When S188/S190 phosphorylation is prevented by mutating the two residues into alanine, several functional consequences can be observed. Cells spread less, have reduced protrusive activity and migrate more actively. Collectively, our data demonstrate for the first time that serine-regulated degradation of paxillin plays a key role in the modulation of membrane dynamics and consequently, in the control of cell motility.

In line with our data, degradation of paxillin by two different mechanisms has already been reported. On one hand, it was shown to depend on a calpain-regulated process [9-11]. On the other hand, ubiquitylation of paxillin was postulated to lead to selective degradation of the focal adhesion-associated insoluble pool but not the cytosolic pool of the protein [13]. This suggests that the ubiquitylated forms of paxillin degraded in the NBT-II cell line used in our study could belong mainly to the adhesive contact-associated pool of the protein. In NBT-II cells, the insoluble pool of paxillin corresponds to approximately 30% of total paxillin (data not shown).

Using a non-phosphorylatable mutant of paxillin at serines 188 and 190, we found that phosphorylation of these sites was necessary for paxillin stabilization by collagen. The mechanism by which this effect is achieved remains elusive. Phosphorylated S188/S190 may constitute a regulated docking site for a protein partner, thereby sequestering paxillin away from the proteasomal machinery of degradation. Alternatively, S188/S190 phosphorylation may prevent other posttranslational modifications of the molecule required for ubiquitylation and/or degradation. In an attempt to investigate the significance of the increased paxillin stability induced by S188/190 phosphorylation on collagen, we demonstrate that phosphorylation on serines 188/190 is not necessary for cell adhesion but is important for cell spreading. It is now accepted that paxillin plays an important role in cell spreading since a number of mutants act in a dominant-negative fashion to reduce this process. CHO.K1 fibroblasts stably expressing a paxillin variant lacking the LD4 motif present a defect in cell spreading on fibronectin [41]. Expression of the ERK/GSK-3 phosphorylation site double mutant, S126/130A, also strongly inhibits cell spreading of paxillin-null fibroblasts on fibronectin [23]. Collectively, these data suggest that paxillin may control cell spreading through multiple signaling pathways.

We also noticed that expression of EGFP-S188/190A paxillin increases cell migration on collagen but not basal cell motility (on plastic), indicating that phosphorylation of serines 188 and 190 regulates collagen-induced but not basal cell migration. To our knowledge, this is the first time that serine phosphorylation of paxillin is shown to be negatively involved in cell motility. These results suggest that the function of paxillin may be orchestrated by an array of negative as well as positive posttranslational modifications. They could be combined on the same molecule leading to an integrated behavior or, alternatively, could arise on molecules localized at different intracellular sites. In the present study, we observe that cells expressing EGFP-S188/190A paxillin mutant spread less but migrate more on collagen than control cells. Reduction in cell spreading induced by the expression of mutant paxillin is consistent with an increase in cell motility, as cells derived from mice deficient in the focal adhesion molecule vinculin likewise show reduced cell spreading and increased cell migration [42].

In addition to negatively regulating cell migration, phosphorylation of paxillin serines 188/190 is important for the collagen-mediated increase in protein stability. In this respect, paxillin ubiquitylation was previously reported to be associated with the inhibition of cell migration [13]. Proteasomal degradation of another focal adhesion protein, FAK, was also shown to inhibit cell migration [12]. Moreover, we observed an inverse correlation between cell migration and membrane protrusion dynamics. In that sense, it is commonly accepted that in order for a cell to translocate properly, membrane protrusions at the leading edge of the cell need to be stabilized, leading to a reduction of protrusion dynamics [43].

Our results also show that a decrease in paxillin stability is associated with a decrease in protrusion dynamics. There are conflicting reports about the role of localized proteolysis at the leading edge of cells in regulating lamellipodia dynamics. Calpain activity was found to be necessary for stabilizing protrusions at the leading edge of migrating cells [9,44]. In contrast, other studies support a role for calpains in limiting membrane extensions [9,45,46], suggesting that calpains may function both as a negative and positive regulator of protrusion events. It is required for stabilization of protrusions at the leading edge while suppressing protrusions in other parts of the cell. Other critical mediators of cell motility, including Rho-pathway components, have a similar relationship between their activities and protrusion stabilization/retraction events, serving both as negative and positive regulators of cell motility [47, 48, 49, 50]. We postulate that proteasome-dependent proteolysis of focal-adhesion molecules such as paxillin may serve similar functions. Degradation of paxillin would be inhibited in stabilized protrusions while taking place in labile membrane extensions, leading to polarization of the cell in the direction of movement. Further experiments are required to explore the role of S188/190 phosphorylation in polarization of cell migration and to establish whether the two serines are equally implicated in these effects.

## Methods

### Reagents and antibodies

Lactacystin, used at 1 μM, was from Calbiochem (Darmstadt, Germany). Monoclonal antibodies against paxillin (clone 349) and ubiquitin (clone P4D1) were from Transduction Laboratories (Lexington, KY, USA) and Santa Cruz Biotechnology (Santa Cruz, CA, USA), respectively. Rat type I collagen was from BD Bioscience (San Jose, CA, USA).

### Plasmids

Complementary DNA coding for chicken S188/190A mutant paxillin in the pcDNA3 expression vector was kindly provided by Dr. C.E. Turner (State University of New York, Syracuse, NY, USA). To construct the EGFP-tagged paxillin S188/190A mutant, the pcDNA3-S188/190A plasmid was subcloned in EGFP-C3 plasmid (Invitrogen, Carlsbad, CA, USA) using BamHI and XbaI restriction sites. The wild-type EGFP-paxillin was previously described [20]. All constructs were sequenced prior to use (MWG-Biotech, Ebersberg, Germany).

### Cell culture and transfection

NBT-II carcinoma cell line was cultured routinely in DMEM (Gibco BRL-Invitrogen, Carlsbad, CA, USA) supplemented with 1% glutamine and 10% heat-inactivated fetal calf serum as previously described [27].

For immunoprecipitation experiments, 1.5 × 10^6 ^cells plated one day before in 60-mm dishes were transfected with 6 μg DNA and polyethylenimine (Sigma, St. Louis, MO, USA) in DMEM for 3 h 30 as described before [28].

For all other experiments, 2 × 10^6 ^cells plated one day before in 60-mm dishes were transfected with Lipofectamine™ 2000 (Invitrogen) following the manufacturer's recommendations.

### Western blots and immunoprecipitations

Cells were trypsinized, resuspended in complete medium for 15 min and replated on plastic dishes or on plates pre-coated overnight at 4°C with collagen (10 μg/ml). For western blotting experiments, cells were lysed with NP-40 buffer (1% NP-40, 50 mM Tris pH 8.0, 150 mM NaCl, 5 mM EDTA), protease inhibitors (Complete tablets, Roche Diagnostics, Mannheim, Germany) and a cocktail of phosphatase inhibitors (1 mM Na-orthovanadate, 1 mM Na-pyrophosphate, 25 mM β-glycerophosphate, 1 mM Na-fluoride). For immunoprecipitations, cells were lysed with Triton buffer (1% Triton X-100, 10 mM Tris pH 7.4, 150 mM NaCl, 1 mM EDTA, Complete proteases and phosphatase inhibitors). Immunoprecipitations were performed on equivalent amounts of proteins as described before [20].

For Western blot analysis of paxillin, equivalent amounts of proteins were separated by SDS-PAGE electrophoresis and transferred onto polyvinylidene membranes (Immobilon P, Millipore Corp., Bedford, MA, USA). Western blot detection of paxillin was performed essentially as described previously [20].

### Ubiquitylation of paxillin

Cells were lysed in 1% Triton X-100, 1% SDS, 20 mM Tris pH7.5, 150 mM NaCl, 1 mM CaCl_2_, 10 mM N-ethylmaleimide, protease and phosphatase inhibitors, diluted 10 times in the same buffer without SDS and 1 mg of proteins was used for paxillin immunoprecipitation. Samples were separated by SDS-PAGE and subjected to Western blotting with an anti-ubiquitin antibody.

### Pulse chase analysis

Non-transfected or cells transfected with plasmids encoding for EGFP-paxillin or EGFP-S188/190A paxillin were harvested by trypsinization and maintained in suspension in complete medium without methionine and cysteine for 30 min at 37°C. One hundred μCi of ^35^S-methionine/cysteine mix (Redivue™ PRO-MIX™, Amersham Biosciences, Piscataway, NJ, USA) was added per 1 × 10^6 ^cells. Cells were then washed 3 times in radioactive-free medium and plated onto plastic or collagen-coated plates and incubated at 37°C for increasing periods of time prior to cell lysis. 1.5 × 10^6 ^cells were lysed and paxillin immunoprecipitation was done as described previously. Proteins were separated by SDS-PAGE electrophoresis, gels were dried and exposed to autoradiographic films (Amersham Biosciences). Quantification of the autoradiographs from three independent experiments was done using Image J free software (NIH, USA). Means and SEM were then calculated. The Student's *t *test was used to evaluate the data.

### Cell proliferation assay

NBT-II cells (7.5 × 10^4 ^cells) were plated in 60-mm culture dishes pre-coated with collagen (10 μg/ml) and allowed to grow at 37°C. Each day, cells were trypsinized and counted using a hemocytometer.

### Cell adhesion and spreading

NBT-II cells transiently expressing EGFP-paxillin or EGFP-S188/190A paxillin were trypsinized, washed 3 times with DMEM and resuspended in 100 μl of DMEM. Eighty thousand cells were then plated on 96 well plates pre-coated overnight at 4°C with increasing concentrations of collagen and saturated for 1 h at room temperature with 5% BSA (Sigma). Non-specific cell adhesion was measured on wells coated with BSA only. After incubation at 37°C for 1 h, wells were washed 3 times with DMEM to remove non-adherent cells. The remaining adherent cells were fixed for 20 min with 3.8% paraformaldehyde, washed extensively and then stained for 10 min with 5% crystal violet in methanol. Plates were then washed with water until the excess dye was removed. Crystal violet incorporated within the cells was released with 100 μl acetic acid (10% v/v) and the absorbance was quantified at 580 nm. All experiments were performed in triplicate with at least three independent experiments done. Means and SEM were then calculated. The Student's *t *test was used to evaluate the data.

For cell spreading assays, NBT-II transfected cells were trypsinized, washed 3 times with DMEM, resuspended at a concentration of 0.5 × 10^6 ^cells/ml. Cells were then plated on 6 wells plates pre-coated with 10 μg/ml collagen. After incubation at 37°C for the indicated times, attached cells were fixed with 3.8% paraformaldehyde for 20 min at room temperature. Round phase bright, refractile cells were scored as unspread. Phase dark, nonrefractile cells and cells that were elongated were defined as spread. Spreading was calculated as the ratio between spread and attached GFP-positive cells. All experiments were done in duplicate with at least three independent experiments performed. Means and SEM were calculated. The Student's *t *test was used to evaluate the data.

### Cell migration assay

Eighteen hours after transfection with EGFP-tagged constructs, 10^5 ^cells were trypsinized and plated on plastic or collagen-coated 35-mm Petri dishes in complete medium. After 4 h, the dish was placed on the motorized stage of a Leica 2D multi-positioning inverted microscope microscopy (Leica Microsystems, Wetzlar, Germany) equipped with a chamber providing controlled temperature and CO_2 _concentration. Phase-contrast and fluorescent images were acquired from a cool SNAP™ CCD camera (PhotoMetrics, Tucson, AZ, USA). Image acquisition was controlled using the Metamorph software (Universal Imaging Corp., Downingtown, PA, USA). Images of the fluorescently tagged cells were acquired at the beginning of the experiment. Phase-contrast time-lapse movies were then recorded at 4-minutes intervals. Motility of individual cells was evaluated by tracking their movement during 10 h using Metamorph software. The average speed of locomotion (μm/h) was calculated as the total track length divided by the number of hours recorded. For each experimental condition, 50–100 GFP-expressing cells were recorded and analyzed. At least three independent experiments were performed for each condition. Means and SEM were calculated. The Student's *t *test was used to evaluate the data.

### Immunofluorescence

NBT-II cells were trypsinized 24 h after transient transfection and plated on glass cover-slips for an additional 24 h. Cells were then fixed with 4% paraformaldehyde for 20 min and permeabilized with 0.1% Triton X-100 for 2 min prior to incubation with 100 μM 4'6-diamidino-2-phenylindole (DAPI, Sigma, St. Louis, MO, USA) for 5 min. The cover-slips were mounted in PBS/glycerol+100 mg/ml 1,4-diazabicyclo [2.2.2]octane (DABCO, Sigma) and observed using a 100× objective by 3D immunofluorescence microscopy (Leica Microsystems). Images were deconvoluted and treated using the Metamorph software.

### Membrane protrusion dynamics

Membrane protrusion analysis was performed using a 40× objective on a 2D multi-positioning inverted fluorescence microscope (Leica Microsystems). Phase-contrast images were captured at 15 sec intervals for 5 min using a Cool SNAP™ CCD camera. In each experiment, at least 5 fields were analyzed per condition. For each condition, a total of 35–50 cells were scored in 5 independent experiments. Membrane protrusion analysis was done using Image J software and based on a program described elsewhere [29] with slight modifications. Briefly, a black mask covering the cell was drawn for every plane in the stack. Binary images in which cell was in black against a white background were obtained using the threshold. Subsequent images in the stack were overlaid with each other and a differential image created. Any pixel undergoing a change in gray value was scored as positive. A binary image was then created in which positive pixels were displayed as black against a white background. The total black pixels for a single cell in the 5-minutes interval were then scored. For each experiment, the average number of pixels displaying a change in gray level was determined. It was converted into change in cell area by taking into account the real size of pixels. Two other parameters were analyzed: the number of protrusions per cell was calculated by adding the number of extensions present at the cell periphery in the 21 images of the entire movie. The protrusive index represents the ratio of the change in cell area divided by the number of protrusions.

## Competing interests

The author(s) declare that they have no competing interests.

## Authors' contributions

NAZ carried out the experiments and wrote the draft manuscript. AMV and BB participated in the experimental design and edited the manuscript.
